# Menthol and flavored tobacco products in LMICs: A growing menace

**DOI:** 10.18332/tid/146366

**Published:** 2022-04-14

**Authors:** Mateusz Zatoński, Karin Silver, Sarah Plummer, Rosemary Hiscock

**Affiliations:** 1 Tobacco Control Research Group, Department for Health, University of Bath, Bath, United Kingdom; 2 Tobacco-Tactics, Tobacco Control Research Group, Department for Health, University of Bath, Bath, United Kingdom; 3 Department for Health, University of Bath, Bath, United Kingdom

**Keywords:** LMIC, menthol, flavor, cigarettes, capsules

## Abstract

**INTRODUCTION:**

High income jurisdictions are banning menthol/flavored cigarettes and other tobacco products because they attract young people and create dependence. This study explores the importance of menthol and other flavored tobacco products for tobacco markets in low- and middle-income countries (LMICs), identifies countries where the menthol/flavor share is particularly high or rapidly growing, and identifies strategies tobacco companies are using to develop menthol/flavor markets.

**METHODS:**

Research involved analysis of menthol/flavor market data from 2005 to 2019, a systematic review of academic literature, and a scoping exercise with our advocate contacts in LMICs.

**RESULTS:**

The median menthol/capsule market share of the cigarette market grew significantly (p<0.05) between 2005 and 2019, both in lower and upper middle-income countries [lower: 2.5% (IQR: 0.5–4.0) to 6.5% (IQR: 3.6–15.9); and upper: 4.0% (IQR: 0.8–9.8) to 12.3% (IQR: 3.5–24.3)]. Countries with both high market share and high market share growth were Russia, Guatemala, Peru and Nigeria. No market data were available on low-income countries, but the academic literature suggested high prevalence of menthol use in Zambia. Tobacco industry strategies underpinning growth of menthol/flavored tobacco use in LMICs included in-store marketing and display, colorful packs and non-conventional flavor names.

**CONCLUSIONS:**

Menthol/flavor tobacco products are a growing problem in LMICs. In addition to menthol/flavor bans, we recommend marketing bans, point of sale display bans and standardized packaging.

## INTRODUCTION

Tobacco use remains persistently high in low- and middle-income countries (LMICs)^1^. Nearly 80% of all smokers live in LMICs and most deaths from tobacco use occur in those countries^2,3^. Although effective progress has been made in LMICs, the tobacco industry is undermining tobacco control in places, and more research and action is required to stop the LMIC tobacco epidemic^4^.

Menthol cigarettes have been found to increase smoking uptake and to make quitting more difficult^5^. Other flavors also appear to play a key role in initiation and progression to dependence, especially among youth^6^. An increasing number of jurisdictions have banned, or intend to ban, menthol/flavored tobacco products. However, bans are more common among high-income countries (HICs), with the tobacco industry still free to sell tobacco with menthol and other flavors (menthol/ flavor) in many LMICs^5,7^.

Flavors, including menthol, can be added to cigarettes, during manufacture, in two ways. First, flavor can be added when the tobacco is blended before the cigarettes are made. Second, a flavored capsule can be added to the filter. The flavor can be activated by crushing the capsule at any time while smoking^8^. A recent review of capsules noted that they are the fastest growing combustible product, have a strong appeal to youth, and more research is needed on their use in LMICs^9^.

In this study, we aim to first understand the patterns of menthol/flavor cigarette market share in LMICs via analysis of market data. Second, we aim to understand how tobacco industry activities relate to these patterns through a systematic review of the existing academic literature on menthol/flavored tobacco use in LMICs, and a scoping exercise with tobacco control advocates in LMICs.

## METHODS

### Understanding the patterns of menthol/flavor market share in LMICs via analysis of market data

An analysis of market data was carried out to understand whether menthol/flavored products are sold in LMICs and, if so, which LMICs have the highest menthol/flavor share of the market, and which have the steepest growth in the menthol/flavor share.

Data were only available for one tobacco product: cigarettes. Euromonitor International collates data from official statistics and trade sources on the market share (percent) of the number (volume) of cigarette sticks sold by country, which have: 1) a flavor capsule (all flavors), 2) are menthol (but do not have a capsule), and 3) neither of these (standard sticks). Cigarettes were defined by Euromonitor as machine manufactured white-stick products and Indonesian kreteks. Bidis, papirosy and hybrid products were excluded. The market consisted of retail sales but excluded duty-free sales and illicit sales. Available years were 2005–2019 at the time of data download (26 December 2020). The combined menthol flavor with no capsule and capsule (menthol/capsule) market share for each country was calculated by subtracting the percentage of standard sticks from 100%.

Countries were classified by Gross National Income (GNI) per capita as defined by the World Bank^10^. Two country income groupings were used for analysis: 1) lower middle-income countries (incomes between US$ 1046–4095); and 2) upper middle-income countries (incomes between US$ 4096–12695)^10^. Median market share and interquartile range in 2005 and 2019 were calculated for the two income groups. Wilcoxon tests were carried out in Excel to assess whether significant change had occurred^11^.

Two groups of countries were further detailed in the analysis. The first were countries with a ‘high market share’ of menthol/flavored products: countries in which the menthol/capsule cigarette market share was greater than 20% of the total market volume in 2019. The second group were countries with ‘high market share growth’ of menthol/flavored products: countries in which the menthol/capsule cigarette market share doubled between 2005–2019 and was continuing to grow for the final three years of the period. Annual median market shares for income groups, and for the high market share growth and high market share country groupings, were calculated and presented as a line graph.

### Understanding the mechanisms which produce observed patterns of menthol market share in LMICs

*Systematic review*

The systematic review’s objectives were first, to identify any further LMICs with high menthol market share not already found via cigarette market data, and second, to understand how the tobacco industry undermines the control of menthol/flavored tobacco with specific reference to LMICs.

*Search strategy*

Studies were identified through two database searches conducted in October 2020. The first search was for conventional tobacco products and the second search was for heated tobacco products (HTPs). This search was exploratory; tobacco companies promoted flavored HTPs to HIC smokers ahead of the EU menthol ban^12^ and we wanted to identify the potential for similar activity in LMICs. The latter search included common acronyms and leading brand names. The two search strings were: 1) [menthol* or flavor* or flavor*] AND [tobacco or cigar*], and 2) [menthol* or flavor* or flavor*] AND [‘heat-not-burn’ OR HTP OR HNB OR IQOS OR ‘heat* tobacco’]. The database searches were conducted using Scopus (since 2016), PubMed (last 5 years), Web of Science (last 5 years), Google Scholar (since 2019 first term, since 2016 second term), and Business Source Complete (since 2016). Time frames reflected available database search settings.

*Inclusion and exclusion criteria*

Inclusion and exclusion criteria were developed via a title search of the first 1000 studies. Each author assessed 250 titles. Articles were included if they focused on the prevalence, patterns of use, and harm perceptions of menthol or flavored cigarette, other flavored combustible tobacco products (including shisha), flavored HTPs; or tobacco industry marketing, promotion or opposition to regulation of these products in LMICs. Articles were excluded if they focused on medical consequences or chemistry of menthol and flavored tobacco products. One author (SP) screened the remaining studies using the inclusion and exclusion criteria, first assessing titles then abstracts and full texts.

*Studies identified*

The initial search generated 6307 studies. There were 3769 studies after duplication removal, 758 after first sift (title), 57 after second sift (abstract). Nine studies (peer reviewed articles and conference abstracts) met the inclusion criteria.

The included studies (Table 1) were set in: the Philippines (n=2); Ethiopia (n=1); Guatemala (n=1); Argentina, Bolivia, Brazil, Chile and Peru (n=1); Kenya and Zambia (n=1); and Brazil (n=3). Study designs were prevalence surveys (n=3), documentary analysis of tobacco control legislation (n=2), observational studies of convenience store marketing (n=2), quantitative content analysis of packs (n=1), and focus groups on packaging of flavored cigarettes (n=1).

**Table 1:** Systematic review study characteristics

*Study reference*	Type*	*Location (date)*	*Sample details*	*Procedures*
13	Conference abstract	Zambia (2014) Kenya (2012)	N=1449 adult smokers from the International Tobacco Control (ITC) Survey	Survey questions on use, choice and, in Zambia only, perceptions, of menthol cigarettes
14	Conference abstract	Brazil: Rio de Janeiro, São Paulo, and Porto Alegre (2016–2017)	N=1216 adult smokers from the International Tobacco Control (ITC) Survey	Survey questions on menthol cigarette use, beliefs about harmfulness and support for banning additives
15	Journal paper	Guatemala (2019)	Convenience stores in mid and high socioeconomic status neighborhoods in Guatemala City (N=60) and Quetzaltenango (N=15)	Checklist to assess the prevalence and characteristics of in-store marketing of capsule cigarettes, e-cigarettes and HTPs
16	Journal paper	Philippines (2016)	75 cigarette packs purchased from supermarkets, stores and kiosks in Manila, Cebu, and Davao	Packs were classified by whether they were flavored and had a capsule and pack design (structure, images, color, and descriptors)
17	Conference abstract	Latin America (2017)	N=1188 retailers located within 100–250 m of 310 primary and secondary schools in one major city in each of Argentina, Bolivia, Brazil, Chile and Peru	Trained observers recorded information about cigarette products displays, advertising and special promotions
18	Journal paper	Philippines (2019)	8 focus groups with 63 young adults (aged 18–24 years) in Manila, stratified by gender and smoking status	Participants ranked cigarette packs on: a) harm, and b) attractiveness. They were asked to discuss flavor imagery and descriptors
19	Journal paper	Brazil (2011)	640 female smokers and non-smokers aged 16–26 years from a market research company consumer panel	Participants were randomly assigned to three experimental conditions to view and rank: a) branded packs, b) plain packs with descriptors, or c) branded packs with no descriptors
20	Journal paper	Brazil (January 2016 to June 2018)	Legislative and related documents, academic literature	Retrospective qualitative review of tobacco industry arguments and strategies to prevent additives ban implementation
21	Journal paper	Ethiopia (1942 to 2018)	Legislative and related documents, academic literature	Qualitative review of documents including analysis of the level of compliance of the National Tobacco Control Directive 2015 and WHO FCTC

* All conference abstracts were peer reviewed for the World Conference of Tobacco or Health in 2018^28^.

*Scoping exercise with tobacco control contacts in LMICs*

An exploratory questionnaire was sent in February 2021 to tobacco control advocates (working for tobacco control non-governmental organizations) linked to our research group. We selected advocates from 13 LMIC countries of interest based on our analysis of market data and our systematic review. The questionnaire consisted of eight open-ended questions on the status of menthol/flavor tobacco products, relevant data sources, and industry activity in each country. Responses were received from ten countries: three in Africa, three in Asia, three in South America and one of four Eastern European countries approached.

*Strategy for identifying tobacco industry interference underlying market share patterns*

Key themes were identified from the systematic literature review articles and agreed between the authors. Responses from the tobacco control advocate scoping questionnaire were grouped according to these themes.

## RESULTS

### The main patterns of menthol/flavor cigarettes use in LMICs identifiable from analysis of market data

The World Bank identifies 55 upper middle-income countries (MICs), 55 lower MICs and 27 low-income countries (LICs)^10^. Euromonitor data on menthol/ capsules included 24 upper MICs, 14 lower MICs, and 0 LICs. The median menthol/capsule market share grew significantly (p<0.05) between 2005 and 2019 among both lower and upper MICs [lower: 2.5% (IQR: 0.5–4.0) to 6.5% (IQR: 3.6–15.9); and upper: 4.0% (IQR: 0.8–9.8) to 12.3% (IQR: 3.5–24.3)]. Thus, a growing menthol/flavor market exists in MICs, with only North Macedonia registering zero market share of menthol/capsule products in 2019.

High menthol/capsule market share and high menthol/capsule market share growth countries are presented in Table 2. There were eight lower MICs and nine upper MICs with high market share growth. There were three lower MICs and seven upper MICs with high market share. There was no geographical pattern. Of particular concern were countries with high market share and high market share growth: Nigeria, a lower MIC, and three upper MICs: Russia, Guatemala and Peru.

**Table 2:** LMICs with high market share and/or high market share growth of menthol/capsule cigarettes by country income

*Country group ^a^*	*High market share ^b^*	*High market share growth ^c^*	*High share and high growth*
Lower middle income	Cameroon, Nigeria, Philippines	Uzbekistan, Bolivia, Egypt, Nigeria, India, Pakistan, Vietnam, Ukraine	Nigeria
Upper middle income	Malaysia, Thailand, Russia, Columbia, Dominican Republic, Guatemala, Peru	Azerbaijan, Kazakhstan, Bosnia and Herzegovina, Russia, Argentina, Costa Rica, Guatemala, Peru, Belarus	Russia, Guatemala, Peru

a Country group is World Bank grouping by Gross National Income (GNI) per capita: lower middle-income countries: US$ 1046–4095; upper middle-income countries: US$ 4096–12695^10^.

b High market share means a market share of 20% or more in 2019.

c High market share growth means market share doubled 2005–2019 and was growing in the last three years.

**Figure 1:**
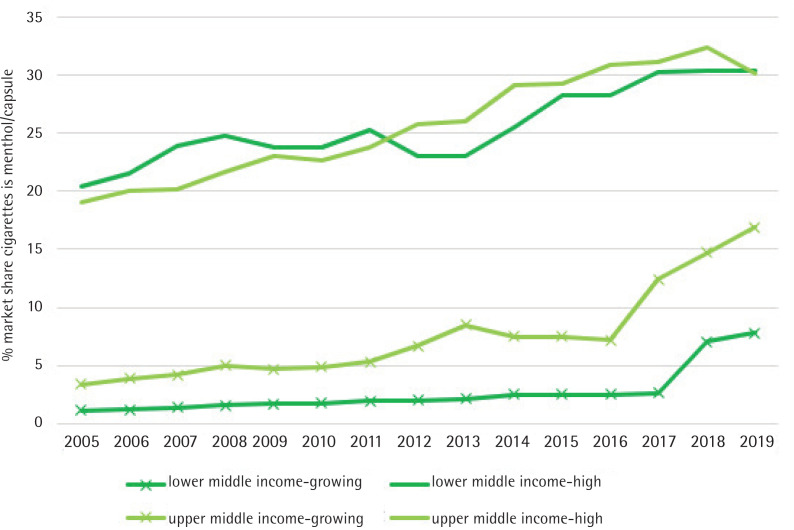
Menthol/capsule cigarette market share among countries with growing and high market share

Market share growth occurred throughout the period for countries with high market share –although there was a plateau for lower MICs 2008–2014 (Figure 1). For countries with high market share growth, however, there appears to be a sharp change in the pace of growth after 2016 for upper MICs and after 2017 for lower MICs. Market share increased faster after 2016–2017.

Analysis of market data implies that menthol/flavor use is present in MICs and that it has grown in recent years. However, no data for LICs were available. The systematic review had potential to identify further LMIC countries where there are concerns about menthol/flavor tobacco use.

### Tobacco industry activity underlying menthol market share patterns

The main themes identified from systematic review articles and scoping exercise with tobacco control advocates were: the presence of a menthol/flavor market, marketing strategies and policy, and industry interference with policy making.

*The presence of a menthol/flavor market in LMICs*

*Evidence from the academic literature*

Five studies described menthol/flavor markets. International Tobacco Control (ITC) survey data revealed two countries (n=1449 smokers) not identified via Euromonitor data with a high prevalence of menthol smokers: Zambia (43%) and Kenya (21%)^13^. Zambian data were not collated by Euromonitor, and Kenyan menthol/flavor market share was recorded by Euromonitor as only 7%. Further insight for Zambia was reported: menthol was most commonly used among younger (69% of survey responses), middle income (36%) and non-daily smokers (42%). ITC data from Brazil (n=1216 smokers) suggested that a comparatively lower percentage (8%) of regular smokers smoked menthol^14^.

Three studies described the market in terms of product availability: in Guatemala, all 75 sampled stores sold flavored products^15^. Philip Morris International, Japan Tobacco International, British American Tobacco, and Korea Tobacco & Ginseng all had flavored capsule cigarettes on the market in the Philippines^16^. Of a sample of 1188 Latin American cigarette retailers located close to primary or secondary schools, 85% sold flavored cigarettes and 71% sold cigarettes with flavored capsules^17^. Menthol flavored HEETS for heated tobacco products (HTPs) were also reported in Guatemala^15^.

*Evidence from the scoping exercise with tobacco control advocates*

Respondents stated that menthol/flavor cigarettes are popular in South America. However, respondents from Asia stated these products were uncommon – unlike flavored smokeless tobacco products. Respondents from Africa and Asia noted both rising use and extensive point-of-sale advertising.

Respondents stated that menthol/flavored tobacco products were available as cigarettes and capsule cigarettes. In addition, menthol/flavored waterpipe tobacco was mentioned. HTPs and nicotine pouches were mentioned by respondents from South America and Eastern Europe.

Menthol/flavored cigarettes were observed as playing an important role in smoking initiation and maintenance among youth and young adults by respondents from South America, Africa and Eastern Europe, with some reporting high use among women. However, it was noted that systematic data on use were not available in some countries.

South American respondents indicated that there is little production of menthol/flavored cigarettes in the region, and they are mostly imported from abroad. Some African respondents, however, suggested that such products are manufactured locally.

### Marketing of menthol/flavored products

*Evidence from the academic literature*

*In-store marketing*

Most (88%) indoor tobacco advertisement in 77 convenience stores in Guatemala were found to be for capsule cigarettes^15^. Cigarette retailers (n=1188) located near schools were observed in cities in five other Latin American countries (Argentina, Bolivia, Brazil, Chile, Peru)^17^. Among retailers that sold flavored cigarettes, 92% had flavored cigarettes on display, typically near cashier zones and confectionary, 34% had advertisements for flavored cigarettes, and 7% had special promotions for flavored cigarettes.

*Package design*

A pack-design study in the Philippines (n=75 packs), found menthol brands had hard packs, giving a quality feel and for capsule protection; brands containing a capsule, had a greater technological appeal than non-menthol packs^16^. Flavored capsule packs were rated by young adult focus group participants (n=63: aged 18–24 years, Philippines) as more attractive^18^. Green colored packs, used for menthol flavored cigarettes, were associated with nature and organics by nonsmokers in the Philippines although green (and red) packs were perceived as more harmful than blue and white^18^.

*Flavor descriptors*

Packs with flavor descriptors were favored over packs with no descriptors by women aged 16–24 years (smokers and non-smokers) (n=640) as part of an online experiment in Brazil^19^. In five Latin American countries, 69% of cigarette retailers (n=1188) sold cigarettes with unconventional descriptors such as ‘Fusion Blast’, ‘Double Click’, and ‘Ruby Ice’^17^. A pack study found sensory descriptors like ‘fresh’ were used for menthol variants in the Philippines^16^. The descriptors ‘light’ and ‘cool’ were perceived as less harmful whereas ‘strong’ was perceived as more harmful by focus group participants also in the Philippines^18^. Thus, harm perception of menthol cigarettes appears to be heavily influenced by vocabulary.

*Harm perception*

Tobacco companies argue that menthol/flavored products are not more harmful than standard tobacco variants, despite independent evidence suggesting the contrary^5^. Thus, it is likely that industry marketing would aim to stop people believing that menthol/ flavors are harmful. Harm perception was included in three survey studies: 36% of smokers in Zambia^13^ and 13% of smokers in Brazil^14^ indicated that menthol cigarettes are less harmful than non-menthol. Additionally, 33% of smokers from Brazil stated that menthols are smoother on the throat and chest than non-menthol cigarettes^14^.

In a young adult (n=63) focus-group study in the Philippines with separate focus groups for men and women, and for smokers and non-smokers, participants were asked to rank a selection of packs by their design. The majority of the rankings and the accompanying discussion indicated that they believed cigarettes could be more or less harmful, basing their judgement on package design and flavor^18^.

*Evidence from the scoping exercise with tobacco control advocates*

Prevalent point-of-sale advertising of flavored tobacco products was mentioned by several respondents. Other advertising sites included branding on stores, shopping malls, billboards, social media and elsewhere online, TV, and magazines. Some also referred to the use of brand ambassadors and stealth marketing. Lack of restriction of advertising of HTPs, while restrictions do exist on conventional tobacco products, was also noted.

### Policy making and interference

*Evidence from the academic literature: Industry interference*

A review of academic, government and commercial documents^20^ found the tobacco industry used strategies to successfully delay menthol/flavor resolutions in Brazil which were created to prohibit the addition of substances that enhance the attractiveness of tobacco products through flavor and taste. Strategies included instigating demonstrations by tobacco farming front groups, media articles, litigation, lobbying, and industry-commissioned studies questioning legislation rationales. Arguments used by the tobacco industry included: a ban threatens employment, increases illicit trade, or prevents successful growing of burley tobacco; there is a lack of evidence that a ban would reduce smoking; and that a ban would be illegal. The authors noted that these strategies had also been used elsewhere. The legislation was delayed for many years despite arguments presented having little scientific basis or evidence.

Ethiopia banned flavored tobacco products including menthol, as part of its first comprehensive tobacco control legislation in 2015, one of the first countries to do so. A review of Ethiopian legislation and implementation^21^ described industry interference but no interference specifically relating to the menthol/flavor ban was mentioned.

*Policy recommendations*

Five studies called for greater action and support banning flavor additives in the Philippines^16,18^, Latin America^17^ and Brazil^14,20^. One study, from Brazil, found that 60% of surveyed smokers supported a complete ban on all cigarette additives, including flavorings^14^.

LMICs with no ban often have other major tobacco control policy gaps that are not necessarily menthol/flavor related^21^ but exacerbate the negative impact of menthol/flavors. Two major such policies are standardized packaging (which can include restrictions on flavor descriptors) and prohibitions of marketing especially near schools^17^.

Governments have more conflicts of interest in tobacco growing areas as they receive much needed foreign currency for tobacco exports^21^ and multinational companies can threaten this income stream if governments attempt to put tobacco controls in place^20^. Thus, LMICs should reduce their reliance on multinationals, and HICs should support and provide alternative ways for LMICs to reduce poverty.

*Evidence from the scoping exercise with tobacco control advocates*

Only one respondent stated that there was regulation of menthol/flavored tobacco products in their country, although several reported that legislation restricting sales was in progress. Respondents from some countries suggested that the tobacco industry has been actively trying to impede legislative progress on the control of menthol/flavored cigarettes, or to undermine existing legislation. Respondents from other countries stated they were not aware of any attempts by the industry to undermine existing legislation.

### Summary

Underlying mechanisms for promoting menthol/flavors in LMICs identified from academic literature included: in-store advertising and point-of-sale display including in retailers near schools; pack design and flavor descriptors; and interference in policy making regarding bans. In addition, tobacco control advocates noted that there was advertising for flavored products on billboards, on TV, online, and via brand ambassadors.

In addition to promoting menthol, policy interference was mentioned in articles and by advocates from several countries. In some LMICs, flavored products are imported rather than made locally. This could potentially stimulate legislation to ban flavors.

## DISCUSSION

The market data analysis highlighted the easy availability of menthol/flavored cigarettes and high and/or growing market share in MICs. The academic literature (nine studies) and survey of ten tobacco control advocates from ten countries confirmed the presence of menthol/flavored tobacco products in LMICs and suggested that there were tobacco industry activities underlying growth. These activities included marketing, particularly in stores and on packaging, and interference in policy making, which reduces the likelihood of restrictions on flavored products, sales or promotion. Our three data sources provided broadly similar findings across LMICs from different regions, and from studies with a variety of designs.

The countries identified as ‘high market share’ and ‘high market share growth’ should be of particular public health concern. A higher proportion of menthol/flavored products implies that new smokers could be more likely to try menthol/flavored cigarettes thus easing the transition to habitual smoking and second, smokers of menthol/flavored cigarettes are less likely to quit^22^. Guatemala, Nigeria, Peru and Russia were identified in the analysis of market data as countries with both high market share and high market share growth of these products.

There appears to be a marked increase in the pace of market share growth after 2016–2017. This was around the time that the EU Tobacco Products Directive became law, with the flavor ban (except menthol) implemented from 2017. Non-menthol flavor use has seen a small decline in the EU after 2017^23^ and menthol cigarette market share began to decline^5^ despite the menthol ban not coming into force until 2020. It is possible that tobacco companies could have increased their marketing of menthol and flavored products in LMIC countries as EU sales were winding down.

Research on the topic remains scarce, as evidenced by the low number of LMIC-focused studies identified in our systematic review. In particular, we know very little about the use of menthol/flavored tobacco products, tobacco industry marketing strategies, or corporate political activities around menthol/flavored tobacco products in LMICs.

### Limitations

Euromonitor International was used as the data source for the menthol/flavor market. Euromonitor is an independent market research company providing global quantitative analysis data on industries, countries and consumers including menthol/flavored cigarettes. However, Euromonitor accepts money from the tobacco industry and tobacco industry funded entities^24^. One grant was explicitly flavor related – measuring the impact of the EU flavor ban^25^. Nevertheless, such companies also require accurate data so we believe the data to be of sufficient quality to use but must be treated with caution.

Conflict of interest statements were only available for five of the nine studies in the systematic review. The tobacco control advocates were previously confirmed not to have conflicts of interest, however it is worth noting that authors did not attempt to verify information from the tobacco control advocate scoping exercise, as it was outside the scope of this project.

The available data on the menthol/flavor market was limited. Menthol/capsule market data were not available for any low-income country and less than half of middle-income countries. Only cigarette data were available, but tobacco control advocates noted that LMICs may have significant use of other flavored tobacco products such as waterpipe, smokeless tobacco and HTPs. Furthermore, only menthol flavored cigarette data were differentiated, unless flavor was added via a capsule. Market share of volume was the only statistic available; data on absolute rather than relative growth, and value of the menthol markets and leading brands, were not available. Reliable data on manufacture and market share support governments to legislate, and evaluate the impact of tobacco control regulations once implemented^26^. A lack of data also hinders advocates in urging governments to act, and in countering tobacco industry interference at country level.

For confidentiality reasons we did not identify tobacco advocates by country, only by continent. There was a limited response from tobacco control advocates from LMICs in Eastern Europe/West Asia and no articles identified from countries in these regions for the review. Further work is required to make country specific recommendations.

Euromonitor suggested menthol/flavors held a low market share in Kenya, but a reviewed study suggested high prevalence of use. This contradiction may reflect the difficulty of collecting data in LMICs and highlights the need for better data collection in future.

### Policy recommendations

Young people in many LMICs have menthol/flavored tobacco products available to them with fewer of the tobacco control policies in place which would limit their attractiveness and misrepresentation of harmfulness. Such policies, now enacted in many HICs, include standardized packaging, point-of-sale display bans and marketing bans. For LMICs, tobacco industry pressure may be harder to counter due to local employment and foreign currency acquired via tobacco farming^27^. Nevertheless, some LMIC governments have some legislation or are attempting to develop legislation. We recommend LMIC governments enact menthol/flavor bans in addition to other tobacco control measures.

## CONCLUSIONS

This review highlights the importance of the regulation of menthol/flavored tobacco products in LMICs. Menthol/flavored tobacco use is high in some LMICs and a growing problem in many others. Tobacco industry promotion of menthol/flavor may have grown recently as HICs put menthol/flavor bans in place. Legislation is needed to restrict the sale of menthol and flavored tobacco products, accompanied by standardized packaging and advertising and display bans in countries where they are not yet in place. Tobacco control researchers and advocates need to understand how such legislation can be supported. Academic research and market data are sparse in LMICs, particularly LICs, the focus being on Europe and North America. More research is needed to help governments monitor the tobacco industry as recommended by WHO.

## Data Availability

The data supporting this research cannot be made available for privacy or other reasons. Euromonitor: our licence does not allow us to share the data. Scoping survey: as the survey was a scoping survey, we did not ask for individual questionnaire responses to be made available. Literature review: the reviewed articles are listed within the references. Open access articles are available online.
